# Month of birth and level of insolation as risk factors for multiple sclerosis in Poland

**DOI:** 10.1371/journal.pone.0175156

**Published:** 2017-04-06

**Authors:** Paweł Dobrakowski, Michał Bogocz, Kamil Cholewa, Mateusz Rajchel, Katarzyna Kapica-Topczewska, Sławomir Wawrzyniak, Halina Bartosik-Psujek, Alina Kułakowska, Dorota Koziarska, Monika Adamczyk-Sowa

**Affiliations:** 1 Department of Neurology in Zabrze, Medical University of Silesia, Zabrze, Poland; 2 Department of Neurology, Medical University of Białystok, Białystok, Poland; 3 Department of Neurology, 10^th^ Military Hospital with Policlinic, Bydgoszcz, Poland; 4 Clinical Department of Neurology, Rzeszow State Hospital No 2, Rzeszow, Poland; 5 Department of Neurology, Pomeranian Medical University, Szczecin, Poland; University of Oxford, UNITED KINGDOM

## Abstract

**Introduction:**

Many studies have shown that people born in the spring are at a higher risk of developing multiple sclerosis (MS). This may be associated with lower levels of sun exposure, and consequently, lower levels of vitamin D3 during pregnancy. However, these relationships have not been verified thus far in any countries in Central Europe.

**Objective:**

The aim of our study was to determine the frequency distribution of births for each calendar month in patients suffering from MS in Poland.

**Methods:**

We analyzed data for 2574 patients diagnosed with MS (1758 women, 816 men) living in Poland for an extended period. We added corrections resulting from the frequency distribution of births for the years in which the patients were born. We applied the Hewitt test for seasonality with Rogerson modification for 3-, 4-, or 6-month pulses or periods. Moreover, we examined the average number hours of sunshine in every month of the year.

**Results:**

The rank-sums for successive 3- and 4-month segments indicated the period from September to December and from October to December as having a significantly lower incidence (*p* = 0.027 and *p* = 0.054, respectively). We did not find a correlation between with hours of sunshine in the first trimester of pregnancy, the child’s birth month, and the child developing MS.

**Conclusions:**

We were able to confirm a seasonal variation in the risk of MS in Poland. However, these findings did not correlate with hours of sunshine during the first trimester of pregnancy.

## Introduction

Multiple sclerosis (MS) is a T-cell mediated complex autoimmune disease, causing central nervous system damage resulting in progressive disability. Precise etiology of MS is unknown, but is postulated to arise from a combination of genetic and environmental factors, which can trigger the disease [1].

An association between birth month and risk of neurological diseases has been well reported. The first report considering MS came from studies in the Netherlands and Japan [2,3].

Level of insolation may influence the maternal and fetal immune system, increasing the risk of offspring developing MS. Higher exposure to UVB radiation and resulting higher serum vitamin D concentration in the first trimester of pregnancy is possibly associated with lower risk of MS [4,5]. A Finnish study showed that insufficient maternal 25-hydroxy vitamin D intake during pregnancy may increase the risk of MS in offspring [6].

The largest pooled analysis of birth timing in MS included 42045 patients, and was performed by Willer et al., based on studies from Denmark, Sweden, Great Britain, and Canada [7].

A number of international studies suggests that in the northern hemisphere, risk of developing MS is higher for those born in the spring and lower for those born in autumn [7–9]. However, in central Eastern Europe, such studies are lacking. In Poland, this topic has not been properly examined to date. This region is of great interest for comparative analysis, because of the great seasonal fluctuation of UV light.

## Materials and methods

**Fig 1:**
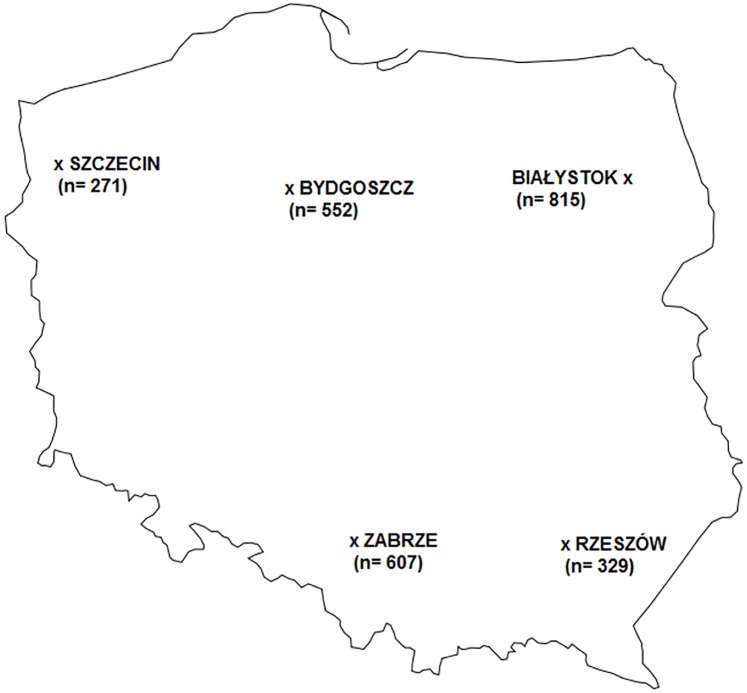
Location of the MS units included in the study (in brackets number of patients).

This study was conducted among patients with MS according to the criteria of Poser and McDonald [10,11]. We obtained cases registered in the MS units in Zabrze and Rzeszów (south of Poland), and Bydgoszcz, Szczecin, and Białystok (north of the country) (Fig 1). The latitudinal extent between cities was from 50.04° to 53.43° N. Information on insolation was obtained from the Institute of Meteorology and Water Management (a national research and development unit). Data were analyzed separately for individual months from 1962 to 1986, which corresponds to the mean age of the study group ± standard deviation. The approval of the Bioethics Committee of Pomeranian Medical University was obtained. We used the anonymized registry data. The clinical information was collected after written informed consent. The information collected did not cause harm to patients.

Demographic and clinical data were analyzed including date of birth, sex, and clinical features of the disease. The birth months in this group were used to calculate the expected birth numbers per month among patients with MS S1 Table. This is a crucial element, because birth month in a normal population varies significantly with respect to geographical location and time.

Patients’ birth dates were compared with birth dates among the general Polish population, as published in the Demographic Yearbook of Poland. Frequency of births remained stable over the entire period under consideration, with a higher frequency of births in spring and a smaller proportion in autumn. Births were analyzed for the year 1976 (median birth year) in smoothed figures, where the monthly average was 100.

The data were analyzed using VassarStats. Seasonality was assessed using the 2 × 2 table chi-square test with Yates’ correction and the generalization of Hewitt test by Rogerson [12–14].

The Hewitt test is a maximal rank sum among all possible rank sums derived using consecutive 6- month periods. In a Rogerson modification, the Hewitt test is extended to include those instances where 3-, 4-, or 5-month pulses or periods of raised incidence are hypothesized.

Month-specific risk of MS was compared with the other 11 months, in terms of odds ratios (ORs) and 95% confidence intervals (CIs).

Differences were considered significant at *p* <0.05.

## Results

We collected data for 2574 patients. The female to male ratio was 2.15:1 (1758 and 816 patients, respectively), and the median birth year was 1976. The mean age was 41.8 ± 11.4.

Total sunshine duration in hours/year was 1581–1816 S1 File.

**Fig 2:**
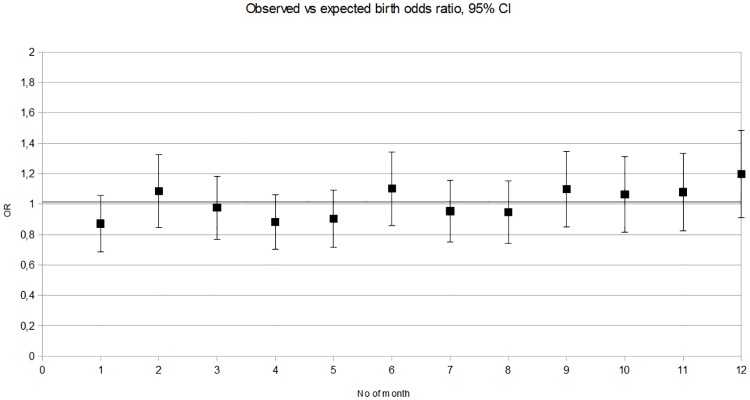
Observed vs expected number of multiple sclerosis births month by month.

At the first glance, we observed that January presented a higher number of MS patients (+13.46%), and December presented a far lower number (-15.53%; Fig 2). However, OR was always near 1 and the *p*-value was >0.05, indicating no rejection of the null hypothesis in the chi-square goodness of fit test. There was no significantly increased risk of MS for any month (Table 1).

**Table 1:** Measured and expected number of births per month (in MS patients) with odds ratio.

Month	MS	SF1976	expected MS	OR (95% CI)	*p- value*
January	246	101	217	0.87 (0.72–1.06)	0.173
February	203	102	219	1.09 (0.89–1.33)	0.446
March	230	105	225	0.98 (0.81–1.18)	0.841
April	262	109	234	0.88 (0.73–1.06)	0.202
May	252	107	230	0.90 (0.75–1.09)	0.315
June	204	104	223	1.10 (0.90–1.34)	0.362
July	235	105	225	0.95 (0.79–1.15)	0.663
August	224	99	213	0.95 (0.78–1.15)	0.617
September	191	97	208	1.10 (0.89–1.35)	0.403
October	184	91	195	1.06 (0.86–1.31)	0.597
November	180	90	193	1.08 (0.87–1.33)	0.517
December	165	91	195	1.20 (0.97–1.48)	0.113

Month: month of birth SF1976: in smoothed figures for 1976—monthly average = 100

expected MS: number of patients expected with MS due to SF1976

The next step was to assess seasonality. The rank-sums for successive 6-month segments indicated that the period from March to August showed a higher incidence of MS (*p* = 0.130). Shorter periods of March to May also showed elevated incidence. However, the corresponding rank-sums (53 and 31, respectively) were not statistically significant according to the Hewitt test (*p* = 0.198).

Additionally, we analyzed periods that showed a lower incidence than expected. For the 4-month period from September to December and the 3-month period from October to December, the difference was significant (*p* = 0.027 and *p* = 0.054 respectively; Table 2).

**Table 2:** Hewitt’s ranks for 6-, 4- and 3-month periods.

6 months			4 months			3 months		
Period	Rank- sum	*p- value*	Period	Rank- sum	*p- value*	Period	Rank- sum	*p- value*
Jan-Jun	52	0.211	Jan-Apr	35	0.591	Jan-Mar	23	0.999
Feb-Jul	51	0.288	Feb-May	36	0.459	Feb-Apr	25	0.959
Mar-Aug	53	0.130	Mar-Jun	37	0.336	Mar-May	31	0.198
Apr-Sep	49	0.374	Apr-Jul	38	0.240	Apr-Jun	29	0.471
May-Oct	40	0.999	May-Aug	33	0.847	May-Jul	26	0.882
Jun-Nov	31	0.581	Jun-Sep	26	1.000	Jun-Aug	22	1.000
Jul-Dec	26	0.211	Jul-Oct	23	1.000	Jul-Sep	20	1.000
Aug-Jan	27	0.288	Aug-Nov	16	0.459	Aug-Oct	14	0.959
Sep-Feb	25	0.130	**Sept-Dec**	**10**	**0.027**	Sep-Nov	9	0.322
Oct-Mar	29	0.496	Oct-Jan	16	0.459	**Oct-Dec**	**6**	**0.054**
Nov-Apr	38	0.999	Nov-Feb	18	0.732	Nov-Jun	13	0.882
Dec-May	47	0.581	Dec-Mar	24	1.000	Dec-Feb	16	0.999

**Fig 3:**
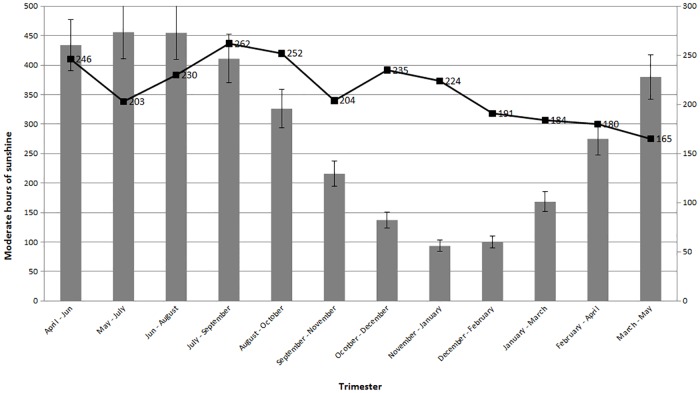
Correlation between insolation in the first trimester of pregnancy and number of SM births.

We have not determined a correlation between insolation in the first trimester of pregnancy and child’s birth month for those that developed MS (R = 0.254; Fig 3).

There were no significant differences between the sexes (*p* = 0.378).

## Discussion

A true birth-month effect in MS carries some attractive implications for potential preventive strategies. Hintzen [15] reminds us about seasonal variation in birth rates in the general population, and that it is common to find a false-positive correlation. Fiddes et al. reasoned that confounding underlies this apparent association of birth-month and MS risk. A well-known limitation of such studies is using a place of living and not of origin in analyses [16]. Interestingly, Poland is the country with the smallest number of foreign citizens in United Europe (<0.1%, according to Eurostat). Thus, it is highly probable that people who are residents of the area were also born there.

Poland seems to be a proper country to conduct the studies such as ours due to the fact of cultural uniformity and consequently similar culinary traditions across Poland.

In Poland after World War II the migration of people from eastern to western regions resulted in mixing of traditions, including food habits.

In the analyzed period local epidemics were not reported in any of the study regions of Poland. Even in the case of an increase in the incidence of e.g. influenza, such an increase was observed in the whole country. The only epidemic within the analyzed period was reported in Wroclaw in 1963 and was related to smallpox. However, Wroclaw was not included in our analysis.

Moreover, Poland has a relatively small latitudinal extent.

A limited number of studies in the Southern hemisphere have frequently reported an increased risk of MS in patients born in autumn and winter that decreases in spring [5]. However, different conclusions were noted in Brazil [17].

According to Torkildsen et al. [18] a summary of most studies from the Northern hemisphere reported a higher incidence of MS in patients born in spring and summer and a lower incidence in autumn and winter months [7–9,19–24]. In a Canadian study [25], seasonal patterns were noted by James [26] and only two studies reported null significant differences [27,28].

According to the most recent studies from the United Kingdom, risk of MS is associated with birth season (high risk in spring, low risk in autumn), particularly in Scotland [19,29,30]. Similar results were found in Tunisia [31]. However, for a Portuguese population, data from a group of 421 patents did not support the seasonality hypothesis [32]. Furthermore, one study from Kuwait showed a peak in MS risk in December [33].

Some authors suggest that birth month is important only in countries with a high population risk of MS, and this effect is hardly observed in countries receiving optimal sun exposure [18,31]. However, it is important to note that hypovitaminosis D may also be an effect of lifestyle or insufficient dietary intake [34].

Our study suggests that in Poland, a deficiency in births of MS patients occurred between September and December, with a nadir in December. These findings were significant when Hewitt test for 4 months and 3 months was used, but not when a chi-square test was used. We also report more MS births in January, April, and May with no significant differences between these months. We found results similar to our own in a study of Polish-only analysis by Cendrowski [35,36].

In conclusion, our study is one of the first to assess the association between birth month and MS incidence in a Central Eastern European population. We were able to confirm a seasonal variation in MS risk in Poland; however, these findings were not easily correlated with insolation during the first trimester of pregnancy.

The limitations of our study are the unknown individual exposure of the pregnant mother to UVB radiation, or the dietary intake of vitamin D.

## Supporting information

S1 File Number of sunny hours month by month (average from 1962 to 1986). (DOC)

S1 Table Year and month of birth patients in this study. (XLS)
