# Supplementary figures and images for: Month of birth and level of insolation as risk factors for multiple sclerosis in Poland
Source: PLoS One. 2017 Apr 6;12(4):e0175156. doi: 10.1371/journal.pone.0175156 (PMC5383232; doi:10.1371/journal.pone.0175156)

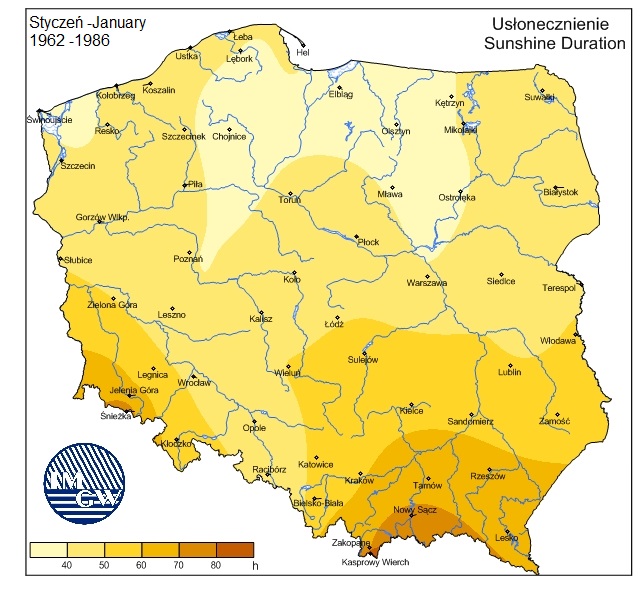

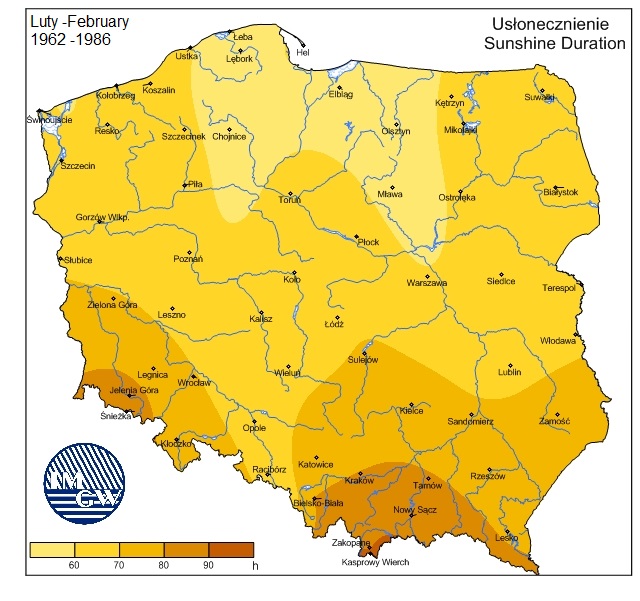


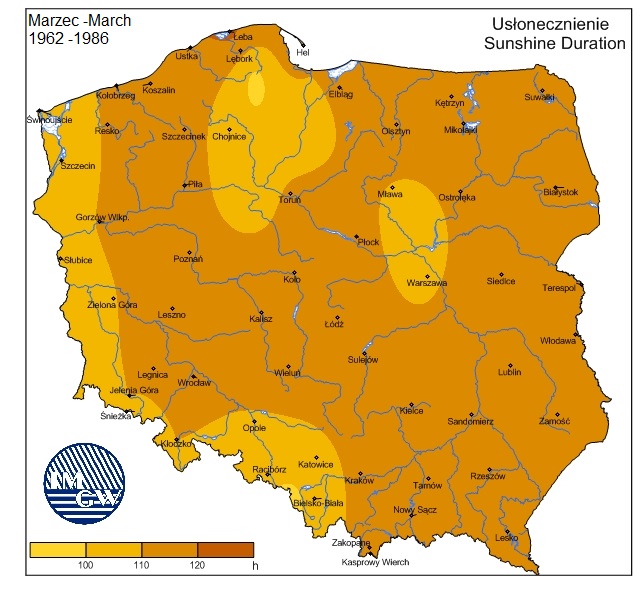

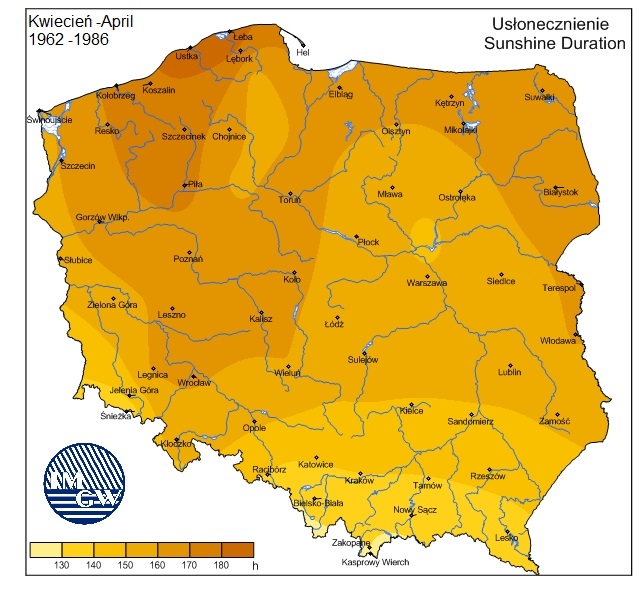


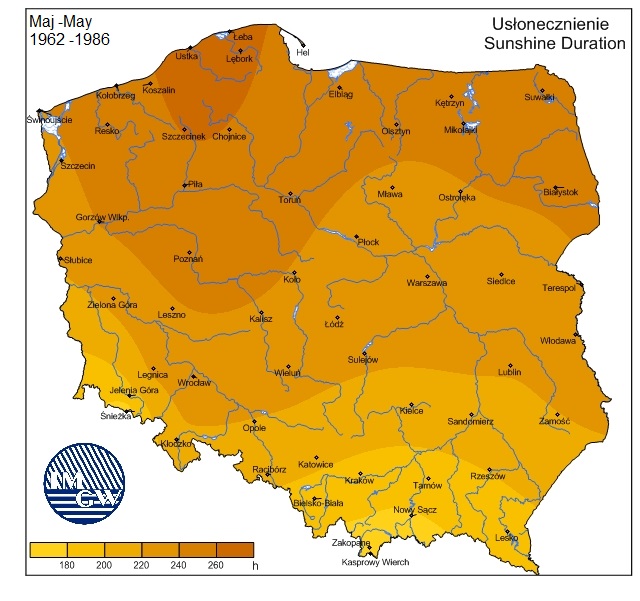

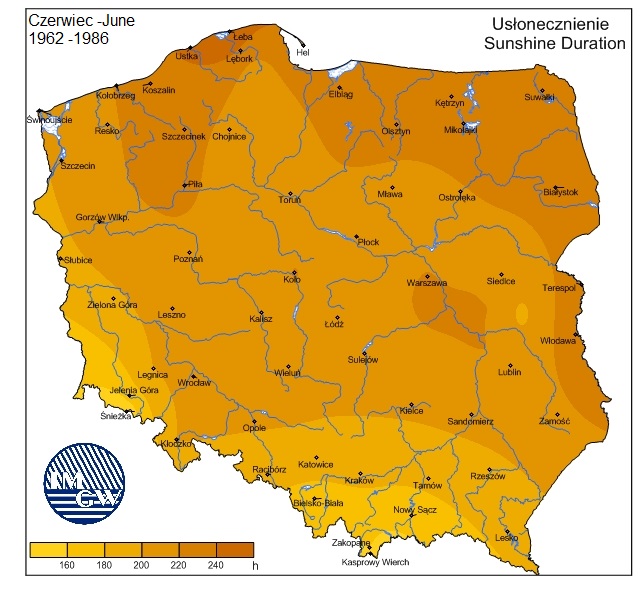


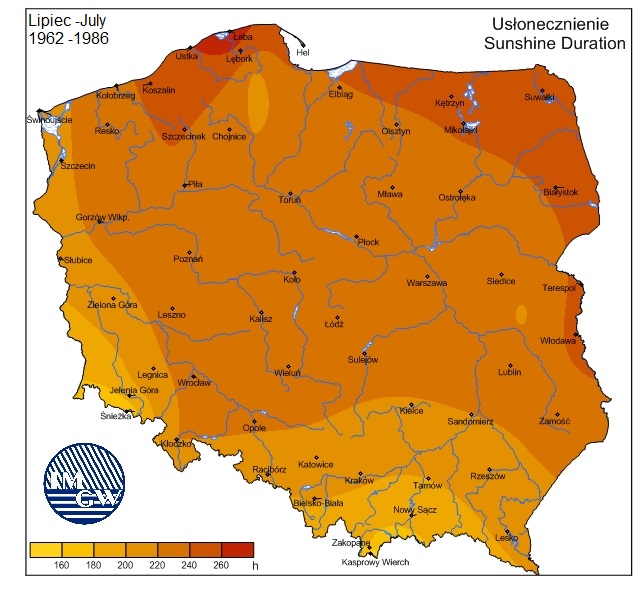

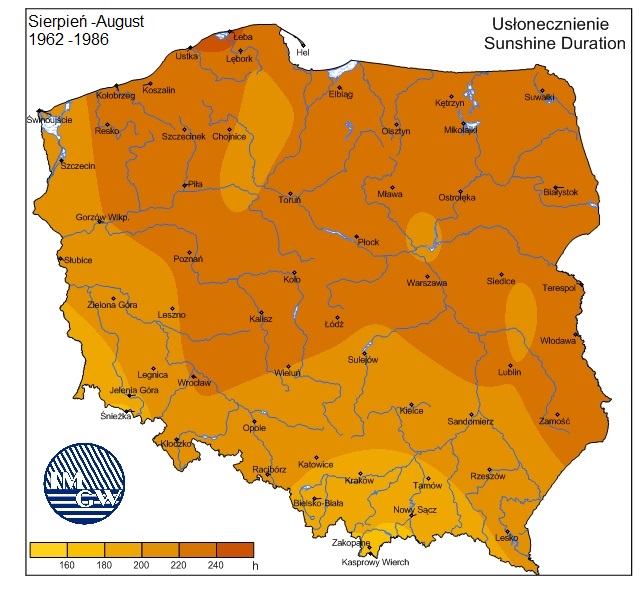


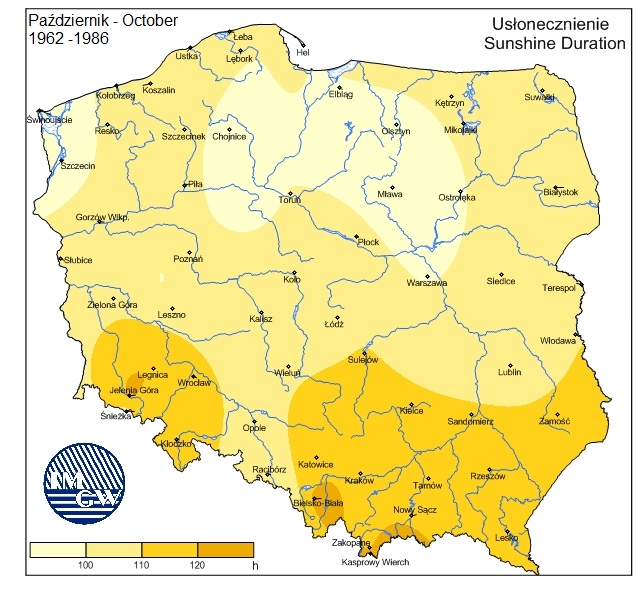

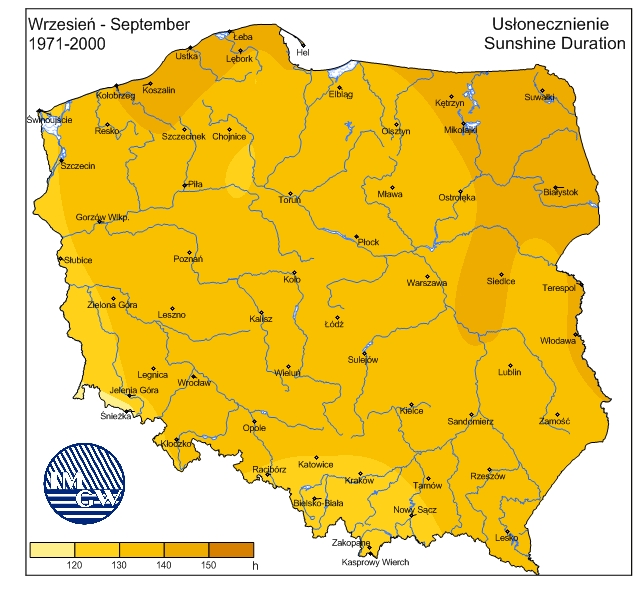


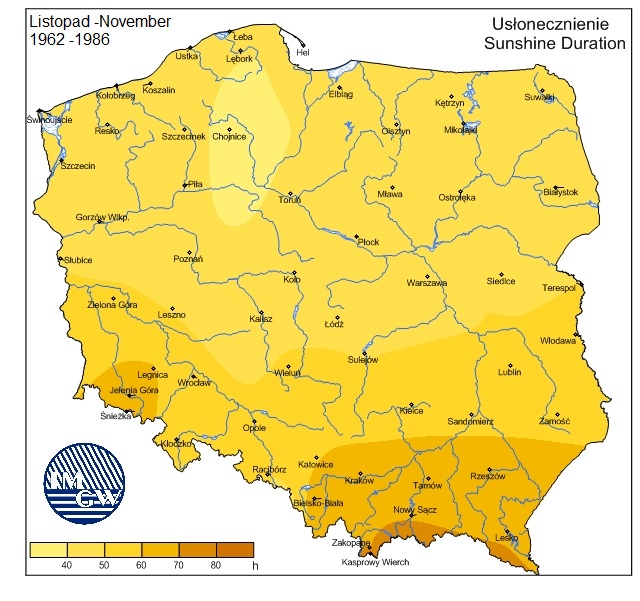

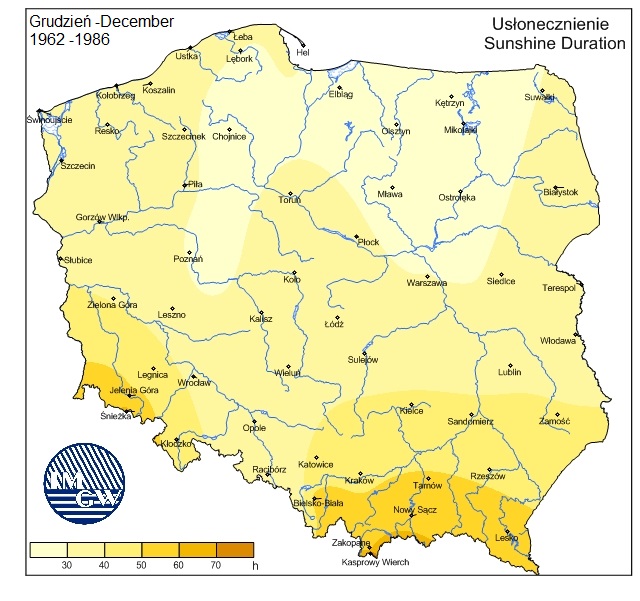

Supplement: S1 File — (DOC) [file pone.0175156.s001.doc]
